# Real-world clinical outcomes of tisagenlecleucel in relapsed or refractory diffuse large B-cell lymphoma: a single-center retrospective study

**DOI:** 10.3389/fonc.2026.1823568

**Published:** 2026-04-24

**Authors:** Gi-June Min, Tong Yoon Kim, Young-Woo Jeon, Sung Soo Park, Silvia Park, Jae-Ho Yoon, Sung-Eun Lee, Byung Sik Cho, Yoo-Jin Kim, Ki-Seong Eom, Hee-Je Kim, Chang-Ki Min, Seok-Goo Cho

**Affiliations:** 1 Department of Hematology, Seoul St. Mary’s Hematology Hospital, College of Medicine, The Catholic University of Korea, Seoul, Republic of Korea; 2 Department of Hematology, Yeouido St. Mary’s Hematology Hospital, College of Medicine, The Catholic University of Korea, Seoul, Republic of Korea

**Keywords:** diffuse large B-cell lymphoma, refractory, relapsed, tisagenlecleucel, treatment outcomes

## Abstract

**Introduction:**

Tisagenlecleucel (Tisa-cel), approved by the Korean Ministry of Food and Drug Safety (MFDS) in 2021 and reimbursed since April 2022, is currently the only second-generation chimeric antigen receptor (CAR) T-cell therapy available in Korea. This study aimed to evaluate the real-world efficacy, safety, logistics, and outcomes of Tisa-cel in Korean patients with relapsed or refractory diffuse large B-cell lymphoma (R/R DLBCL) and identify prognostic factors associated with survival.

**Methods:**

We retrospectively analyzed 79 patients with R/R DLBCL who received Tisa-cel at the Catholic Hematology Hospital between April 2022 and January 2025.

**Results:**

The median decision-to-infusion and vein-to-vein times were 49 and 40 days, respectively. Bridging chemotherapy was administered to 64 patients, with an overall response rate (ORR) of 40.6% and a complete response (CR) rate of 14.1% before infusion. Three months after Tisa-cel treatment, the ORR and CR rates were 72.9% and 64.4%, respectively. Tisa-cel was administered as third-line therapy in 50.6% of the patients, and 48.1% had refractory disease. Of the patients who experienced relapse (43%), approximately half transitioned to palliative care, 8.8% were enrolled in clinical trials, and 5.9% proceeded to allogeneic transplantation. Non-relapse mortality was 11.7%, primarily due to infections (n = 7) and immune effector cell–associated neurotoxicity syndrome (ICANS)-related events (n = 2). Cytokine release syndrome occurred in 70.9% of patients (grade ≥ 3 in 22.8%) and ICANS in 21.5% (grade ≥ 3 in 8.8%), with a median onset at 2 and 5 days, respectively. Tocilizumab and dexamethasone were used in 45.6% and 13.9% of the patients, respectively. With a median follow-up of 11.6 months, the median progression-free survival was 4.9 months, and the median overall survival was 21.6 months. Achieving CR at 3 months was strongly associated with superior survival (p < 0.001).

**Discussion:**

Tisa-cel demonstrated favorable efficacy and manageable safety in Korean patients with R/R DLBCL. Early CR strongly predicted long-term outcomes, whereas ICANS occurrence and steroid use were associated with poorer prognosis, underscoring the need to optimize toxicity management.

## 1 Introduction

Before the advent of chimeric antigen receptor (CAR) T-cell therapy, outcomes in patients with relapsed or refractory (R/R) diffuse large B-cell lymphoma (DLBCL) were dismal. Complete response (CR) rates were only 7–9%, and overall survival (OS) rarely exceeded one year, as consistently reported in both international and Korean retrospective studies (1, 2). In contrast, CD19-directed CAR T-cell therapies, such as axicabtagene ciloleucel (Axi-cel), lisocabtagene maraleucel (Liso-cel), and tisagenlecleucel (Tisa-cel), have markedly improved outcomes in R/R DLBCL, achieving overall response rates (ORRs) of 50–70%, CR rates of 30–50%, and median OS beyond 1 year (3–6). Real-world studies involving older and more heterogeneous patient populations have demonstrated outcomes comparable to those observed in pivotal clinical trials, with similar response and survival rates (7–10). Based on this evidence, the NCCN Guidelines now recommend CAR T-cell therapies and bispecific antibodies (BsAbs) as standard options for patients with R/R DLBCL after two or more prior lines of therapy. Accumulating evidence supports the use of BsAbs in patients who relapse after CAR T-cell therapy, positioning them as important options following transplantation or CAR T-cell failure (11).

In Korea, Tisa-cel remains the only commercially available CAR T-cell therapy since its approval in April 2022. This approval was supported by the pivotal JULIET trial, with a median follow-up of 40.3 months, which demonstrated durable clinical efficacy, including an ORR of 53%, median progression-free survival (PFS) of 2.9 months, and median OS of 11.1 months (6, 12). Adverse events of special interest were clinically manageable; grade 3–4 cytokine release syndrome (CRS) was observed in 22% of the patients, with no CRS-related deaths. With extended follow-up, no new safety signals were identified, confirming the durable efficacy and favorable safety profile of Tisa-cel (6, 12). To date, only a single real-world study evaluating Tisa-cel in R/R DLBCL has been reported in Korea, involving 96 patients treated at a single institution (13). The study reported an ORR of 55.1% at 3 months, with median PFS and OS of 4.5 and 13.9 months, respectively, and grade ≥3 CRS observed in 14.6% of patients (13). In light of the limited real-world data, we present the clinical outcomes and safety profile of Korean adults with R/R DLBCL treated with standard-of-care Tisa-cel over a 2.7-year period at a single academic medical center.

## 2 Materials and methods

### 2.1 Study design and patient population

This retrospective single-center study was conducted at Seoul St. Mary’s Hospital, a tertiary referral institution in Korea. We analyzed 79 adult patients (≥18 years) with R/R DLBCL who received Tisa-cel between April 2022 and January 2025. All patients had pathologically confirmed DLBCL. Pathologic specimens were independently reviewed by two expert hematopathologists who were blinded to clinical outcomes, and diagnoses were established based on tissue morphology and immunohistocKa76hemical findings according to the WHO classification criteria (14). Patients with active CNS involvement and those who received investigational products or off-label CAR T-cell therapies were excluded. Re-biopsy at the time of relapse or refractoriness was performed when clinically feasible, but was not mandatory for all patients, due to the urgency of treatment initiation or anatomical inaccessibility. Patients with CD19-negative disease were excluded from treatment. Baseline demographic and clinical characteristics at the time of CAR T-cell infusion, including age, sex, performance status, disease stage, International Prognostic Index (IPI) score, and prior treatment history, were collected from electronic medical records. Clinical and laboratory data were obtained at diagnosis, pre-apheresis, and 1, 3, 6, and 12 months after Tisa-cel infusion. Assessed variables included Eastern Cooperative Oncology Group performance status (ECOG-PS) and lactate dehydrogenase (LDH), ferritin, and immunoglobulin G (IgG) levels. Hypogammaglobulinemia was defined as a serum IgG level ≤400 mg/dL, and a normal LDH level was defined as ≤250 U/L.

The study protocol was reviewed and approved by the Institutional Review Board (IRB) and Ethics Committee of the Catholic Medical Center, Republic of Korea (approval number: KC22TISI0531). This study was conducted in accordance with the principles of the Declaration of Helsinki. Owing to the retrospective study design and minimal risk to participants, the requirement for informed consent was waived by the IRB, as the study did not adversely affect the rights or welfare of the participants.

### 2.2 CAR T-cell treatment and response evaluation process

All patients underwent a standardized CAR T-cell treatment protocol consisting of eight sequential steps: consultation, baseline evaluation, leukapheresis, manufacturing (with or without bridging chemotherapy), lymphodepletion, CAR T-cell infusion, in-hospital management of early adverse events, and outpatient-based long-term follow-up. During the consultation phase, the patients and their families received detailed counseling and provided written informed consent. Baseline evaluation included laboratory testing and imaging studies to assess disease status and eligibility prior to leukapheresis. Peripheral blood mononuclear cells were collected by leukapheresis and transferred to a Good Manufacturing Practice (GMP)-compliant facility for CAR T-cell production. Bridging chemotherapy was administered when clinically indicated, primarily in patients with bulky residual disease, rapidly progressive disease, or a high tumor burden. Lymphodepletion was induced using fludarabine plus cyclophosphamide or bendamustine.

CAR T-cell infusion was administered following standard premedication, followed by continuous clinical monitoring. Patients were monitored in an inpatient setting for early toxicities, particularly CRS, immune effector cell–associated neurotoxicity syndrome (ICANS), and cytopenias; most were considered eligible for discharge approximately 2 weeks after infusion. All patients received CAR T-cell infusion in an inpatient setting with close monitoring, and a dedicated caregiver was required to ensure safe outpatient monitoring after discharge. During hospitalization, patients were supported by an integrated nursing care system, and after discharge, patients and their caregivers received structured education for early recognition of adverse events and prompt access to medical care. CRS and ICANS were graded according to the American Society for Transplantation and Cell Therapy (ASTCT) criteria, and the highest grade observed during the clinical course was recorded for analysis (15). Blood-to-vein time was defined as the interval from the consultation date to the day of Tisa-cel infusion, whereas vein-to-vein time was defined as the interval from the date of leukapheresis to the day of Tisa-cel infusion. In the later phase, management focused on infection-related complications, prolonged cytopenia, and hypogammaglobulinemia. During long-term follow-up, patients received prophylactic antimicrobial agents and routine vaccinations and were monitored for relapse or disease progression to inform subsequent treatment decisions. All treatment-related adverse events were evaluated according to the National Cancer Institute Common Terminology Criteria for Adverse Events (NCI-CTCAE), version 5.0 (16).

Treatment response was assessed before and after bridging therapy, and at 1-, 3-, and 6-months following Tisa-cel infusion, using contrast-enhanced computed tomography (CT) scans of the neck, chest, and abdomen/pelvis, together with 18F-fluorodeoxyglucose positron emission tomography/computed tomography (FDG PET/CT) according to the Lugano 2014 (International Working Group) response criteria (17). The efficacy endpoints included CR, PR, stable disease (SD), progressive disease (PD), PFS, and OS. ORR was defined as the best response—either CR or PR—achieved at any time after Tisa-cel infusion and before disease progression or the initiation of any new systemic therapy.

### 2.3 Statistical analysis

Descriptive statistics were used to summarize the baseline demographics, disease characteristics, and adverse event incidence. Categorical and continuous variables were compared using the Chi-square test (or Fisher’s exact test, as appropriate) and Student’s *t*-test (or the Wilcoxon rank-sum test), respectively. OS was defined as the time from Tisa-cel infusion to death from any cause, whereas PFS was defined as the time from infusion to documented disease progression or death from any cause. OS and PFS were estimated using the Kaplan–Meier method, with median survival times and corresponding 95% confidence intervals calculated using the product-limit estimator when applicable. The cumulative incidence method was applied to estimate the cumulative incidence of relapse (CIR) and non-relapse mortality (NRM). CIR was defined as the time from Tisa-cel infusion to relapse or progression, and NRM as the time to death without prior relapse. Competing events were treated accordingly, and group comparisons were performed using Gray’s test. Multivariable analyses for OS and PFS were conducted using the Cox proportional hazards regression model, with covariates selected based on established prognostic factors from previous studies (7–10). To minimize the risk of overfitting, the number of covariates included in the multivariable models was restricted relative to the number of observed events. Covariates were selected based on clinical relevance and evidence from prior studies. The Fine–Gray proportional hazards regression model was used to estimate hazard ratios (HRs) for cumulative incidence outcomes. All statistical analyses were performed using R software version 4.3.1 (R Foundation for Statistical Computing, Vienna, Austria). All p-values were two-sided, and a p-value < 0.05 was considered statistically significant.

## 3 Result

### 3.1 Baseline characteristics

Among the 79 patients with R/R DLBCL who received Tisa-cel, the median age at infusion was 61 years (range, 20–83 years), and 57.0% were aged ≥60 years. Most patients had a good performance status (ECOG-PS 0–1, 86.1%), and the majority had advanced-stage disease (stage III–IV, 75.9%). Half of the cohort (51%) had received two prior lines of therapy, and nearly one-third (30.4%) had undergone autologous stem cell transplantation (ASCT). Prior to Tisa-cel infusion, 51.9% of the patients had relapsed disease, 36.7% had relapsed/refractory disease, and 11.4% had primary refractory disease. Pre-infusion laboratory findings showed elevated lactate dehydrogenase (LDH) and ferritin levels and thrombocytopenia in 52.4%, 74.7%, and 67.1% of patients, respectively. Bridging chemotherapy was administered to 64 patients, most commonly with polatuzumab, bendamustine, and rituximab (Pola-BR, 42.2%), followed by bendamustine and rituximab (BR, 28.1%), and other regimens, such as EPOCH (n=12), radiotherapy (n=3), R-DHAP (n=2), and selinexor (n=1). While Pola-BR served as the primary bridging chemotherapy regimen, the absence of reimbursement for polatuzumab in Korea rendered it cost-prohibitive for some patients. As a result, those facing financial constraints received BR alone or other alternative chemotherapy regimens. Radiotherapy was mainly administered to patients with localized lesions, whereas selinexor was given to a patient with secondary CNS lymphoma who was not eligible for systemic chemotherapy. Among the 64 patients who underwent bridging therapy, 40.6% (n=26) achieved CR/PR—most commonly with Pola-BR—and among the 59 of 79 patients assessable at three months after CAR T infusion, lack of response to bridging therapy was significantly associated with failure to achieve CR (p=0.018). The baseline characteristics and responses to bridging chemotherapy prior to CAR T-cell infusion are summarized in **Table 1**.

**Table 1:** Baseline characteristics and response to bridging chemotherapy prior to CAR T-cell infusion (n = 79; 3-month response evaluable in 59 patients).

Characteristics	Total (n=79)	3M non-CR (n=21)	3M CR (n=38)	p
Age, median (range)	61.0 (20 – 83)			0.591
Age ≥60	45 (57.0%)	11 (52.4%)	23 (60.5%)	
Sex				0.060
Male	44 (55.7%)	15 (71.4%)	17 (44.7%)	
Female	35 (44.3%)	6 (28.6%)	21 (55.3%)	
ECOG-PS				0.699
0-1	68 (86.1%)	19 (90.5%)	32 (84.2%)	
2-3	11 (13.9%)	2 (9.5%)	6 (15.8%)	
Stage at diagnosis				1.000
Stage I-II	19 (24.1%)	6 (28.6%)	11 (28.9%)	
Stage III-IV	60 (75.9%)	15 (71.4%)	27 (71.1%)	
Pathology, Cell of origin^*^				0.413
ABC	50 (63.3%)	16 (76.2%)	23 (60.5%)	
GCB	28 (35.4%)	5 (23.8%)	14 (36.8%)	
Richter transformation	1 (1.3%)	0 (0.0%)	1 (2.6%)	
Number of prior treatments				0.106
<3 lines	40 (50.6%)	9 (42.9%)	25 (65.8%)	
≥3 lines	39 (49.4%)	12 (57.1%)	13 (34.2%)	
Prior ASCT				0.571
No	55 (69.6%)	15 (71.4%)	23 (60.5%)	
Yes	24 (30.4%)	6 (28.6%)	15 (39.5%)	
Disease status before CAR T				0.232
Relapsed	41 (51.9%)	9 (42.9%)	25 (65.8%)	
Relapsed Refractory	29 (36.7%)	10 (47.6%)	11 (28.9%)	
Primary refractory	9 (11.4%)	2 (9.5%)	2 (5.3%)	
LDH, Pre-CAR T, median (range)	253 (138-5,000)			0.096
Normal	39 (49.4%)	10 (47.6%)	27 (71.1%)	
Elevated	40 (50.6%)	11 (52.4%)	11 (28.9%)	
Ferritin, Pre-CAR T, median (range)	1,868 (17-13,280)			0.773
Normal	20 (25.3%)	7 (33.3%)	11 (28.9%)	
Elevated	59 (74.7%)	14 (66.7%)	27 (71.1%)	
Platelet, Pre-CAR T, median (range)	98K (3K-368K)			1.000
Normal	26 (32.9%)	7 (33.3%)	14 (36.8%)	
Thrombocytopenia	53 (67.1%)	14 (66.7%)	24 (63.2%)	
Bridging Chemotherapy (n=64)				0.294
Pola-BR	27 (42.2%)	6 (35.3%)	15 (50.0%)	
BR	18 (28.1%)	4 (23.5%)	9 (30.0%)	
Others**	19 (29.7%)	7 (41.2%)	6 (20.0%)	
Bridging responses (n=64)^§^				0.018
CR/PR	26 (40.6%)	5 (29.4%)	20 (66.7%)	
SD/PD	38 (59.4%)	12 (70.6%)	10 (33.3%)	

ASCT, autologous stem cell transplantation; CAR T-cell, chimeric antigen receptor T cell; CR, complete remission; ECOG PS, Eastern Cooperative Oncology Group Performance Status; LDH, lactate dehydrogenase; PD, progressive disease; Pola-BR, polatuzumab vedotin-bendamustine and rituximab; PR, partial remission; SD, stable disease.

*High-risk biologic features were observed in a subset of patients, including MYC rearrangement in 4 patients (5.1%), double expressor lymphoma in 14 (17.7%), double-hit lymphoma in 1 (1.3%), and triple expressor lymphoma in 4 (5.1%).

**Other bridging chemotherapy included EPOCH (n=12), RT (n=3), R-DHAP (n=2), and Selinexor (n=1).

§ Bridging therapy was administered to 64 patients, yielding a CR/PR rate of 40.6%. However, due to attrition at the 3-month response assessment, the number of evaluable patients for chi-square analysis was smaller than the total number of patients who received bridging therapy.

Cell of origin was determined using the Hans algorithm.

Double expressor lymphoma was defined by MYC ≥40% and BCL2 ≥50% expression by immunohistochemistry.

Double-hit lymphoma was defined by MYC and BCL2 rearrangements detected by FISH.

### 3.2 Treatment flow and clinical outcomes

**Figure 1:**
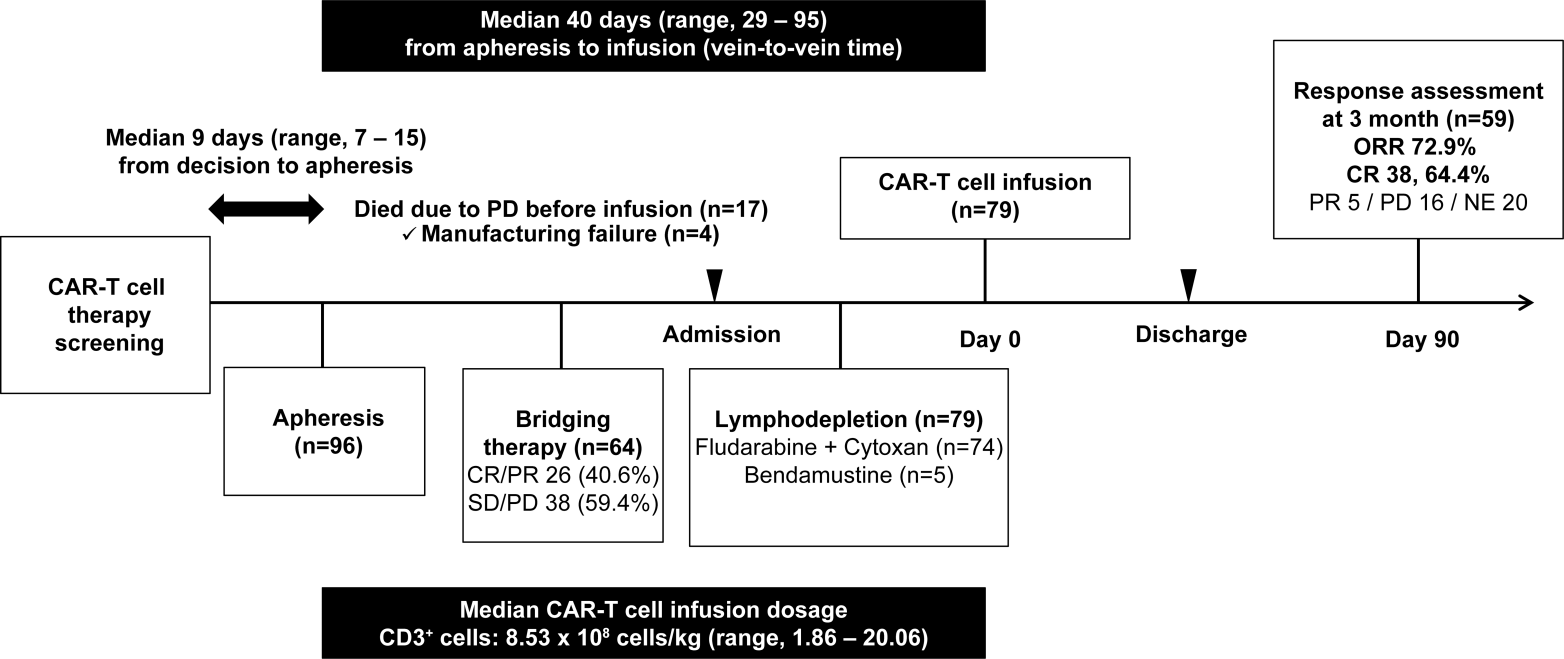
Treatment flow and outcomes of patients receiving Tisa-cel. The median time from the treatment decision to apheresis was 9 days (range, 7–15 days), with a median vein-to-vein interval of 40 days (range, 29–95 days). Among the 96 apheresed patients, 17 died from disease progression, including 4 with manufacturing failure, leaving 79 who received Tisa-cel. Bridging therapy was administered to 64 patients, of whom 40.6% achieved CR/PR. All infused patients underwent lymphodepletion (fludarabine/cyclophosphamide, n=74; bendamustine, n=5), with a median infused dose of 8.53 × 10^8^ CD3^+^ cells/kg (1.86–20.06). At 3 months, 59 evaluable patients had an ORR of 72.9% and a CR rate of 64.4%, with relapse and sepsis-related mortality occurring in 4 (10.5%) and 1 (2.6%) patients, respectively. CAR T-cell, chimeric antigen receptor–T cell; CR, complete remission; NE, not evaluated; ORR, overall response rate; PD, progressive disease; PR, partial remission; SD, stable disease.

The median interval from treatment decision to apheresis was 9 days (range, 7–15 days), and the median vein-to-vein time, defined as the time interval between leukapheresis and Tisa-cel infusion, was 40 days (range, 29–95 days). Of the 96 patients who underwent apheresis, 17 died due to disease progression before infusion, including 4 patients with manufacturing failure. Ultimately, 79 patients (82.3%) received Tisa-cel. All infused patients underwent lymphodepletion—74 with fludarabine/cyclophosphamide and 5 with bendamustine—with a median Tisa-cel dose of 8.53 × 10^8^ CD3^+^ cells/kg (range, 1.86–20.06). Among the 59 evaluable patients at 3 months post-infusion, the ORR was 72.9% (n=43), including a CR rate of 64.4% (n=38). Of the 38 patients who achieved CR, 4 (10.5%) experienced relapse and 1 (2.6%) died from septic shock. The treatment flow and clinical outcomes of patients who received Tisa-cel are shown in **Figure 1**.

### 3.3 Safety outcomes after Tisa-cel infusion

CRS was observed in 70.9% of patients (n=56), with most cases limited to grade 1 (39.2%, n=31) or grade 2 (29.1%, n=23); grade 3 and grade 4 events were uncommon, occurring in 8.9% (n=7) and 1.3% (n=1) of patients, respectively. ICANS occurred in 21.5% of the patients (n=17) and was predominantly low-grade, whereas grade 3 and grade 4 ICANS were reported in only 6.3% (n=5) and 2.5% (n=2) of the patients, respectively. Tocilizumab was administered to 45.6% of patients (n=36), most frequently as one to two doses (25.3%, n=20), while 20.3% (n=16) required three or more doses. Dexamethasone was used in 13.9% of the patients (n=11), primarily for 1–6 days (10.1%, n=8), with prolonged administration (≥7 days) required in 3.8% (n=3). Among the 79 patients, 24 (30.4%) experienced infection-related complications. A total of 24 patients (30.4%) experienced documented infections following CAR T-cell infusion. Infection severity ranged from grade 1 to grade ≥3 according to CTCAE (version 5.0), with clinically significant events such as septic shock. Viral upper respiratory infections occurred in 10 patients, predominantly grade 1–2, except for two cases of COVID-19 pneumonia (grade ≥3). Enterocolitis of unknown etiology (grade 2) occurred in five patients. Bacteremia (grade 3) occurred in four patients, two of whom progressed to septic shock. Two patients developed herpes zoster (grade 2). CMV disease (grade 3) was identified in two patients (one CMV colitis and one CMV pneumonia). Hypogammaglobulinemia (serum IgG ≤400 mg/dL) developed in 23 patients (41.8%) at a median of 4.2 months (range, 1.8–9.7 months) following Tisa-cel infusion. Infection rates did not differ significantly between patients with IgG >400 mg/dL and those with IgG ≤400 mg/dL (45.8% vs. 54.2%, p=0.336). Similarly, the occurrence of infection was not significantly associated with IVIG administration (54.2% with no IVIG vs. 45.8% with IVIG; p=0.630).

### 3.4 Survival outcomes after Tisa-cel infusion

**Figure 2:**
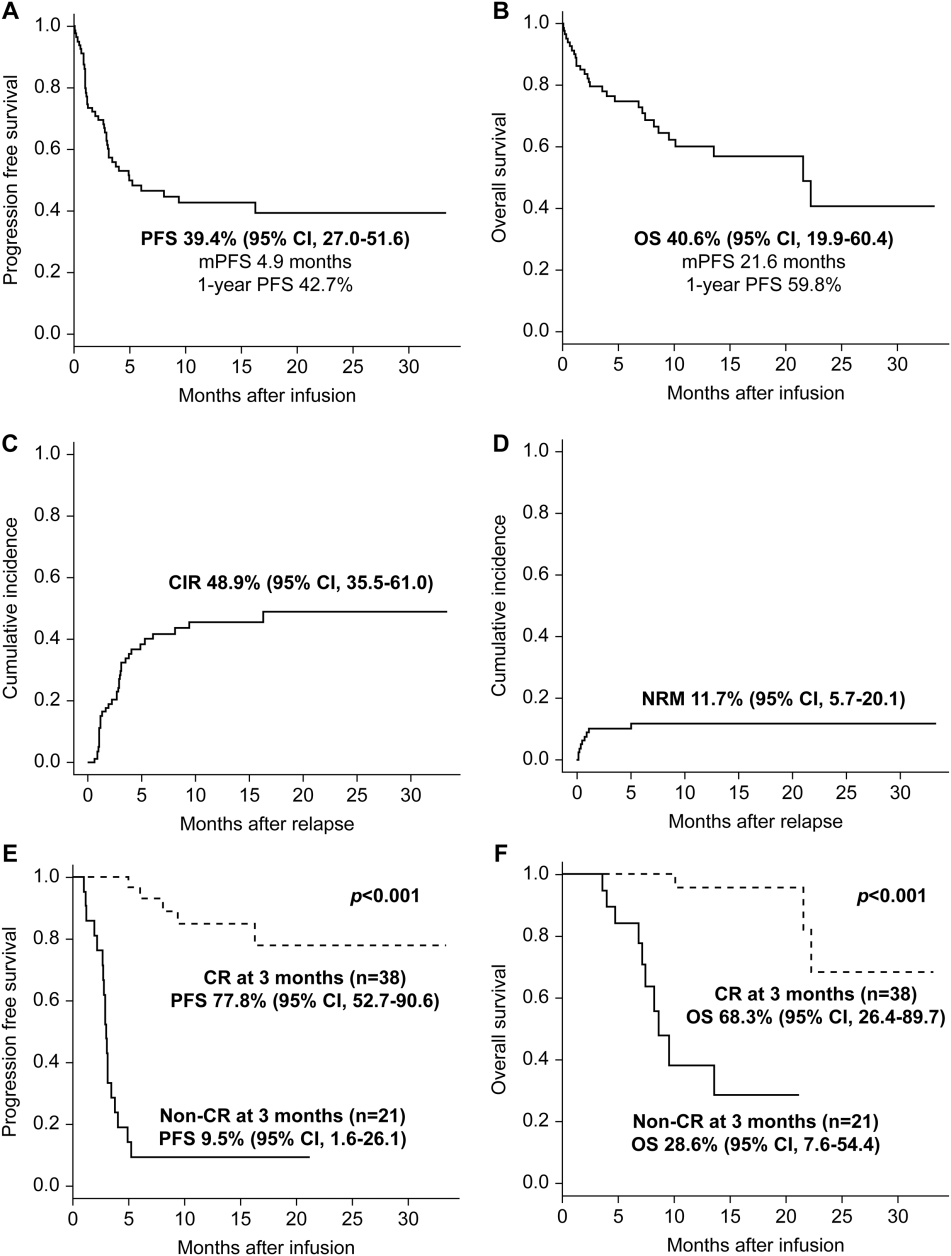
Survival outcomes, relapse, and early response–based stratification after Tisa-cel infusion. **(A)** Kaplan–Meier estimates of PFS for the entire cohort. The median PFS and 1-year PFS rates are shown with 95% CIs. **(B)** Kaplan–Meier estimates of OS for the entire cohort, with median OS and 1-year OS rates. **(C)** CIR after Tisa-cel infusion. **(D)** NRM after Tisa-cel infusion. **(E)** PFS stratified by response status at 3 months after Tisa-cel infusion, comparing patients who achieved CR with those without CR. **(F)** OS stratified by response status 3 months after Tisa-cel infusion. Kaplan–Meier methods were used for OS and PFS analyses, and cumulative incidence functions were applied for CIR and NRM. Patients achieving CR at 3 months demonstrated significantly superior PFS and OS compared with non-CR patients (log-rank p<0.001 for both). Shaded areas or error bars indicate 95% CIs, where applicable. Tisa-cel, tisagenlecleucel; CI, confidence interval; CIR, cumulative incidence of relapse; CR, complete response; NRM, non-relapse mortality; OS, overall survival; PFS, progression-free survival.

At a median follow-up of 11.6 months (range, 3.1–33.4), the median PFS was 4.9 months, with a 1-year PFS rate of 42.7% (**Figure 2A**). The median OS was 21.6 months, and the 1-year OS rate was 59.8% (**Figure 2B**). The CIR and NRM values were 48.9% and 11.7%, respectively (**Figures 2C, D**). Relapse occurred in 34 of 79 patients, of whom 55.9% transitioned to hospice care, 29.4% received conventional chemotherapy, 8.8% participated in clinical trials, and 5.9% underwent allogeneic transplantation. NRM was observed in 9 patients (11.7%), primarily due to infection (77.8%, n=7), with the remainder attributable to ICANS (22.2%, n=2). Seven patients died from infection-related complications following CAR T-cell infusion. Two deaths were attributed to COVID-19, two to bacterial septic shock, two to atypical pneumonia of unknown etiology, and one to CMV pneumonia during ICANS. Patients achieving CR at 3 months after infusion (n=38) demonstrated markedly superior outcomes compared with those without CR. The 1-year PFS was 77.8% versus 9.5% (p<0.001), and the 1-year OS was 68.3% versus 28.6% (p<0.001) in the CR and non-CR groups, respectively (**Figures 2E, F**). The clinical courses of patients unevaluable at the 3-month time point, primarily due to early progression or death, are detailed in **Supplementary Table 1**. Importantly, these patients were included in survival analyses, as overall survival was defined from the time of infusion, thereby maintaining an intent-to-treat framework.

In univariate analyses, fewer prior lines of therapy (two vs. ≥3) were associated with improved OS and PFS and lower NRM. Patients with relapsed disease before Tisa-cel infusion had superior OS and lower CIR than those with refractory disease. Normal pre-infusion LDH levels were associated with improved PFS; however, OS did not differ substantially between the groups. A favorable response to bridging therapy (CR/PR) was strongly associated with improved OS and PFS and a reduced risk of relapse. Conversely, the occurrence of ICANS and the use of dexamethasone were associated with inferior OS and PFS, and ICANS was associated with increased NRM. Achieving CR 3 months after infusion consistently showed the strongest association with favorable survival outcomes and a markedly lower CIR. The detailed results of the univariate analyses are summarized in **Supplementary Table 2**.

**Figure 3:**
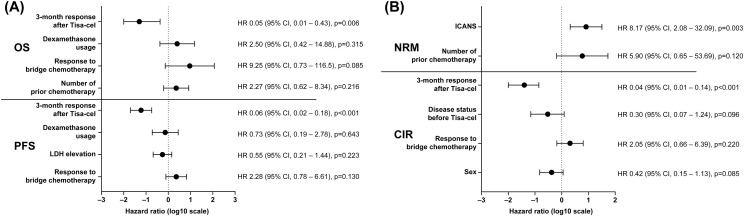
Multivariate analyses of survival outcomes, relapse, and non-relapse mortality after Tisa-cel infusion. **(A)** Forest plots depicting multivariable Cox proportional hazards models for OS and PFS. Variables were selected based on clinical relevance and univariate analyses. Achievement of CR at 3 months after Tisa-cel infusion (CR vs. non-CR) was independently associated with a significantly reduced risk of death and disease progression. HRs are displayed on a log10 scale, with horizontal bars representing 95% CIs. **(B)** Forest plots showing multivariable Fine–Gray proportional subdistribution hazards models for NRM and CIR. ICANS was independently associated with an increased risk of NRM, whereas achievement of CR at 3 months was independently associated with a significantly reduced risk of relapse. HRs are presented on a log10 scale with 95% CIs. Variables not reaching statistical significance are shown for completeness. CI, confidence interval; CIR, cumulative incidence of relapse; CR, complete remission; HR, hazard ratio; ICANS, immune effector cell–associated neurotoxicity syndrome; NRM, non-relapse mortality; OS, overall survival; PFS, progression-free survival; Tisa-cel, tisagenlecleucel.

In multivariate analyses adjusting for clinically relevant variables, achieving CR at 3 months after CAR T-cell infusion remained the strongest independent predictor of favorable outcomes, being associated with significantly improved OS (hazard ratio [HR] 0.05, 95% CI 0.01-0.43; p=0.006) and PFS (HR 0.06, 95% CI 0.02-0.18; p<0.001), as well as a markedly reduced risk of CIR (HR 0.04, 95% CI 0.01-0.14; p<0.001). In contrast, the presence of ICANS was independently associated with increased NRM (HR 8.17, 95% CI 2.08–32.09; p=0.003). Other factors, including sex, number of prior chemotherapy lines, response to bridging chemotherapy, disease status before CAR T-cell therapy, pre-infusion LDH levels, and dexamethasone use, did not retain independent prognostic significance for OS, PFS, or CIR in the multivariate models. The results of the multivariate analysis are shown in **Figure 3**.

## 4 Discussion

When comparing Tisa-cel outcomes across global clinical trials and real-world cohorts, our center demonstrated acceptable efficacy and safety. Large datasets, such as the CIBMTR registry and meta-analyses, report ORRs of 58–60% and CR rates of 39–45% (9, 18). In contrast, our cohort achieved an ORR of 72.9% and a CR rate of 64.4%, which compared favorably with both the meta-analysis (57.7%/39.0%) and the pivotal JULIET trial (52–53%/39–40%), and aligned with results from Japan (73%/55%) and San Diego (67%/53%) (6, 8–10, 12). Beyond confirming the reproducibility outcomes from pivotal trials, this study highlights real-world treatment dynamics, including patient attrition between apheresis and infusion and the role of bridging therapy, in a healthcare system with restricted access to novel agents. The median PFS in our cohort was 4.9 months, numerically longer than that reported in the JULIET trial (2.9 months), and comparable to SMC data (4.3 months) (12, 13). The 1-year PFS rate of 42.7% was higher than that of SMC (33.3%) and approached the 67.0% event-free survival reported in Japan (8, 13). Our median OS was 21.6 months, longer than that reported in JULIET (11–12 months), the meta-analysis (11.7 months), and SMC (13.9 months), and was closer to outcomes from the San Diego cohort (28.4 months) (9, 10, 12, 13). The 1-year OS rate of 59.8% was also comparable to that of Japan (46.3%) and SMC (55.2%) (8, 13).

The safety profile of our cohort was also acceptable. The incidence of grade 3–4 CRS was 22.8%. While this rate is numerically higher than those reported in the CIBMTR (6.0%) and SMC (14.6%) cohorts, it remains within a manageable range. The relatively higher incidence of grade 3–4 CRS observed in our cohort may reflect differences in patient selection, exposure to bridging therapies, or institutional grading practices rather than a true biological difference (13, 18). Grade 3–4 ICANS occurred in 8.8%, consistent with rates from other global datasets. Overall, these findings suggest that Tisa-cel can be administered safely with appropriate supportive care and proactive toxicity management.

The favorable clinical outcomes observed in our center may be partially explained by optimized bridging strategies to reduce disease burden, a higher proportion of patients with normalized pre-infusion LDH, and increasing institutional experience with CAR T therapy. These findings may also reflect effective disease control during bridging, as treatment response rather than the specific regimen was the primary determinant of survival. Although the median follow-up in our study was relatively short (11.6 months), our results fall within the upper range of global experience and support the real-world application of Tisa-cel in relapsed/refractory DLBCL, particularly in Korean academic settings. However, longer follow-up and validation in larger cohorts are required to determine whether these early benefits are sustained over time. Detailed comparative results are summarized in **Supplementary Table 3**.

Infectious complications are common following CAR T therapy, primarily due to prolonged cytopenia, hypogammaglobulinemia, and delayed B- and T-cell reconstitution. In the JULIET trial, IgG, IgA, and IgM levels declined by 74%, 49%, and 63%, respectively, with 20% of all infections occurring between days 60 and 90, and up to 37% of patients requiring IVIG within 5 years (12, 19). B-cell aplasia contributes to persistent hypogammaglobulinemia, with only ~50% of patients recovering B cells within 1 year and approximately two-thirds normalizing immunoglobulin levels within 2 years (20). Concurrently, neutropenia, lymphopenia, and thrombocytopenia may persist for 6–12 months, further impairing immune recovery and increasing susceptibility to infections. Given these prolonged immune deficits, close monitoring of infections is essential, particularly during the early post-infusion period. Although the use of IVIG remains non-standardized, current guidelines recommend routine monitoring of immunoglobulin levels and individualized replacement to maintain IgG levels >600 mg/dL with the goal of reducing infection risk in high-risk patients (21).

Despite the favorable efficacy of CAR T-cell therapy, several challenges limit its long-term outcomes. Antigen loss (e.g., CD19 downregulation), upregulation of inhibitory receptors, lack of costimulatory signals, impaired CAR T expansion and persistence, and T-cell exhaustion all contribute to treatment failure or relapse (22, 23). Recent efforts have focused on defining the molecular determinants of CAR T-cell resistance and developing diagnostic frameworks, including key mechanisms such as antigen loss, T-cell exhaustion, and tumor microenvironment–mediated immune evasion. Recent reviews have also proposed integrated approaches to better characterize resistance patterns and guide subsequent therapeutic strategies (24). More than half of patients eventually experience relapse or disease progression following CD19 CAR T-cell therapy, with reported relapse rates ranging from 30% to 60%; real-world data indicate that approximately 63% of patients relapse or progress within the first year, highlighting post–CAR T management as a major unmet need (25, 26). In a large real-world cohort, outcomes after CAR T-cell failure remained poor, with a median overall survival of 8 months despite subsequent therapy (25). Although antigen loss is a well-described mechanism, clinical series indicate that most relapsed patients retain CD19 expression; however, conventional chemotherapy provides limited benefits. In contrast, novel agents such as polatuzumab- or lenalidomide-based regimens yielded higher response rates, including complete remission, in a subset of patients (25). Among emerging therapies, polatuzumab-based combinations and bispecific antibodies have shown particularly encouraging efficacy, with bispecific antibodies achieving ORRs of approximately 50–60% and CR rates in 30–40% of cases, positioning them as leading options in the post–CAR T setting (25, 27). Allogeneic transplantation can provide long-term survival in a small subset of patients who achieve remission, while novel approaches such as CD22/dual-target CAR T and next-generation cellular therapies are under active investigation (28, 29). Overall, optimal sequencing after CAR T failure remains undefined, highlighting the need for personalized strategies and prioritization of clinical trial enrollment.

This study has several limitations. Its retrospective, single-center design may introduce selection bias and limit generalizability. The relatively small sample size (n = 79) and limited follow-up duration may reduce statistical power and preclude a comprehensive assessment of long-term outcomes and delayed toxicities. Patients with active CNS involvement were excluded due to reimbursement restrictions, representing an important limitation, as outcomes and neurotoxicity profiles such as ICANS could not be evaluated in this high-risk subgroup. In addition, the lack of translational analyses limits mechanistic insights into treatment response and resistance. As Tisa-cel is the only reimbursed CAR T-cell therapy in Korea, comparative analyses with other CAR T products were not feasible. Notably, the median vein-to-vein time was 40 days, and a subset of patients failed to proceed to infusion, primarily due to rapid disease progression, including those with manufacturing failure. These findings emphasize the need for improved patient selection and resource optimization in real-world CAR T-cell therapy.

Nevertheless, despite the availability of multiple CAR T products for DLBCL in other regions, understanding the clinical impact of Tisa-cel remains particularly important in healthcare settings where treatment options are restricted. Therefore, the findings of our study provide meaningful insights into the real-world performance of Tisa-cel and highlight the need for continued efforts to optimize patient selection, improve toxicity management, and extend access to cellular immunotherapy. These considerations are essential for informing clinical practice and guiding strategies to improve outcomes in patients with R/R DLBCL.

In this real-world study, Tisa-cel demonstrated strong efficacy and manageable safety in Korean patients with R/R DLBCL. Achieving a complete response at 3 months emerged as the strongest predictor of survival in our cohort. In addition to this landmark response, early post-infusion laboratory dynamics may further refine risk stratification, as suggested by prior studies (30), and warrant further investigation. Although corticosteroid use was associated with poorer survival outcomes in univariate analyses, this association did not remain significant in multivariable models, suggesting that steroid administration likely reflects underlying disease severity or treatment-related toxicities rather than being an independent prognostic factor. These findings support consideration of steroid-sparing strategies, including early incorporation of anakinra (31), to mitigate neurotoxicity while preserving CAR T-cell function. Future studies incorporating biomarker-driven analyses and prospective multicenter designs are warranted to address these limitations and further refine patient selection and risk-adapted treatment strategies.

## Data Availability

The datasets presented in this article are not readily available due to privacy concerns regarding patient data. Requests to access the datasets should be directed to beichest@nate.com.
