# Supplementary material for: Real-world clinical outcomes of tisagenlecleucel in relapsed or refractory diffuse large B-cell lymphoma: a single-center retrospective study
Source: Front Oncol. 2026 Apr 24;16:1823568. doi: 10.3389/fonc.2026.1823568 (PMC13152773; doi:10.3389/fonc.2026.1823568)
Supplement: Supplementary file 1 [file Table1.docx]

Supplementary Material

**Supplementary Table 1. Clinical courses of patients not evaluable at 3 months (n = 20)**

| **No.** | **Sex/Age** | **Prior lines of treatment** | **Disease status** | **Bridging** | **Bridging response** | **Time from infusion (days)** | **1 mo. response** | **Final outcomes** | **Cause of death** |
| --- | --- | --- | --- | --- | --- | --- | --- | --- | --- |
| 1 | F/64 | 4 | Refractory | BR | PD | 50 | PD | Died | Disease progression |
| 2 | M/20 | 4 | Primary refractory | Pola-BR | SD | 39 | PD | Died | Disease progression |
| 3 | M/67 | 2 | Refractory | - | PD | 36 | PD | Died | Disease progression |
| 4 | M/71 | 2 | Relapsed | BR | PR | 34 | PD | Alive | Hospice care |
| 5 | F/69 | 5 | Relapsed | BR | PD | 32 | CR | Died | Atypical pneumonia |
| 6 | M/55 | 2 | Primary refractory | Pola-BR | PD | 31 | PD | Died | Disease progression |
| 7 | M/71 | 3 | Refractory | DHAP | PD | 31 | PD | Died | Disease progression |
| 8 | F/26 | 3 | Primary refractory | Pola-BR | PD | 30 | PD | Died | Disease progression |
| 9 | F/49 | 2 | Relapsed | Pola-BR | SD | 30 | PD | Died | Disease progression |
| 10 | M/59 | 4 | Refractory | EPOCH | SD | 29 | PD | Died | Disease progression |
| 11 | F/63 | 3 | Relapsed | - | PD | 27 | - | Died | Bacterial septic shock |
| 12 | M/83 | 4 | Refractory | EPOCH | SD | 26 | PD | Died | Disease progression |
| 13 | M/73 | 4 | Relapsed | BR | PD | 26 | PD | Died | Disease progression |
| 14 | M/61 | 3 | Refractory | Pola-BR | PD | 20 | - | Died | ICANS grade 4,  CMV pneumonia |
| 15 | M/24 | 3 | Refractory | EPOCH | SD | 18 | PD | Died | Disease progression |
| 16 | M/52 | 2 | Primary refractory | Pola-BR | SD | 15 | - | Died | ICANS grade 4 |
| 17 | F/69 | 3 | Relapsed | RT | SD | 10 | - | Died | Bacterial septic shock |
| 18 | F/49 | 3 | Primary refractory | EPOCH | PD | 6 | - | Died | COVID-19 |
| 19 | F/76 | 3 | Relapsed | - | SD | 4 | - | Died | Atypical pneumonia |
| 20 | M/40 | 3 | Refractory | BR | PD | 3 | - | Died | COVID-19 |

BR, bendamustine plus rituximab; CR, complete response; DHAP, dexamethasone, high-dose cytarabine, and cisplatin; EPOCH, etoposide, prednisone, vincristine, cyclophosphamide, and doxorubicin; ICANS, immune effector cell–associated neurotoxicity syndrome; PD, progressive disease; Pola-BR, polatuzumab vedotin plus bendamustine and rituximab; PR, partial response; RT, radiotherapy; SD, stable disease.

Time from infusion was calculated from the date of Tisa-cel infusion to the last clinical assessment or death.

Patients listed in this table were excluded from the 3-month response analysis due to early death, rapid disease progression, or inability to undergo response evaluation.

Causes of death were categorized based on clinical assessment, including disease progression, infection, and treatment-related toxicity (e.g., ICANS).

**Supplementary Table 2. Univariate analysis of survival outcomes in CART-cell therapy received patients (Entire cohort, n=79)**

| **Variables** | **OS** | **p** | **PFS** | **p** | **CIR** | **p** | **NRM** | **p** |
| --- | --- | --- | --- | --- | --- | --- | --- | --- |
| **Age (years)** |  | 0.759 |  | 0.599 |  | 0.830 |  | 0.556 |
| <60 (n=34) | 50.6% (30.2-67.9) |  | 48.4% (30.6-64.2) |  | 42.7% (25.2-59.2) |  | 8.8% (2.2-21.3) |  |
| ≥60 (n=45) | 20.8% (1.2-57.3) |  | 30.4% (13.8-48.8) |  | 55.5% (33.2-73.0) |  | 14.1% (5.6-26.6) |  |
| **Sex** |  | 0.767 |  | 0.159 |  | 0.015 |  | 0.164 |
| Male (n=44) | 40.2% (15.4-64.1) |  | 32.9% (18.0-48.6) |  | 60.3% (41.4-74.8) |  | 6.8% (1.7-16.9) |  |
| Female (n=35) | 39.5% (9.1-69.8) |  | 49.3% (30.5-65.7) |  | 32.8% (16.5-50.2) |  | 17.9% (7.0-32.7) |  |
| **ECOG-PS** |  | 0.621 |  | 0.358 |  | 0.121 |  | 0.439 |
| 0-1 (n=68) | 41.1% (20.0-61.3) |  | 36.9% (24.3-49.5) |  | 52.4% (38.3-64.8) |  | 10.7% (4.6-19.6) |  |
| 2-3 (n=11) | 54.5% (16.0-81.9) |  | 63.6% (29.7-84.5) |  | 18.2% (2.4-45.8) |  | 18.2% (2.5-45.6) |  |
| **Ann-Arbor stage at diagnosis** |  | 0.053 |  | 0.126 |  | 0.355 |  | 0.324 |
| Stage I-II (n=19) | 54.9% (9.1-86.0) |  | 52.6% (24.9-74.4) |  | 42.1% (15.8-66.7) |  | 5.3% (0.3-22.0) |  |
| Stage III-IV (n=60) | 35.0% (13.7-57.4) |  | 35.1% (21.9-48.5) |  | 51.1% (36.0-64.4) |  | 13.8% (6.4-24.2) |  |
| **Number of prior Chemotherapy** |  | 0.001 |  | 0.037 |  | 0.798 |  | 0.013 |
| <3 lines (n=40) | 48.0% (9.9-79.2) |  | 45.5% (25.3-63.7) |  | 52.0% (29.7-70.3) |  | 2.5% (0.2-11.4) |  |
| ≥3 lines (n=39) | 31.3% (11.8-53.2) |  | 32.3% (17.6-47.9) |  | 46.5% (29.1-62.2) |  | 21.2% (9.7-35.7) |  |
| **Prior ASCT** |  | 0.536 |  | 0.264 |  | 0.897 |  | 0.164 |
| No (n=55) | 40.6% (15.6-64.6) |  | 36.2% (21.2-51.3) |  | 48.5% (31.5-63.6) |  | 15.3% (7.0-26.6) |  |
| Yes (n=24) | 41.2% (9.4-71.7) |  | 47.8% (26.6-66.3) |  | 48.0% (26.0-67.1) |  | 4.2% (0.3-18.0) |  |
| **Disease status before CAR T** |  | 0.019 |  | 0.036 |  | 0.026 |  | 0.872 |
| Relapse (n=41) | 49.1% (19.0-73.8) |  | 48.8% (29.2-65.8) |  | 38.5% (19.9-56.9) |  | 12.7% (4.5-25.3) |  |
| Refractory (n=38) | 37.8% (19.0-56.5) |  | 27.6% (12.9-44.4) |  | 61.9% (41.1-77.2) |  | 10.5% (3.3-22.7) |  |
| **Platelet** |  | 0.099 |  | 0.246 |  | 0.481 |  | 0.473 |
| Normal (n=53) | 34.1% (9.1-61.6) |  | 36.2% (22.5-50.0) |  | 50.2% (34.6-63.8) |  | 13.6% (5.9-24.6) |  |
| Thrombocytopenia (n=26) | 52.5% (17.5-78.9) |  | 49.4% (27.0-68.3) |  | 42.9% (21.0-63.3) |  | 7.7% (1.3-22.1) |  |
| **LDH** |  | 0.013 |  | <0.001 |  | 0.054 |  | 0.076 |
| Normal (n=39) | 44.7% (17.2-69.3) |  | 53.5% (32.8-70.4) |  | 40.7% (21.4-59.3) |  | 5.8% (1.0-17.3) |  |
| Elevated (n=40) | 46.9% (28.4-63.5) |  | 25.3% (12.4-40.5) |  | 57.2% (38.7-71.9) |  | 17.5% (7.6-30.8) |  |
| **Ferritin** |  | 0.509 |  | 0.237 |  | 0.919 |  | 0.067 |
| Normal (n=20) | 31.1% (1.8-71.2) |  | 49.2% (23.0-71.0) |  | 50.8% (22.6-73.4) |  | 0% |  |
| Elevated (n=59) | 43.9% (21.0-64.8) |  | 37.4% (24.2-50.5) |  | 46.9% (32.6-60.1) |  | 15.7% (7.6-26.4) |  |
| **Bridge chemotherapy (n=64)** |  | 0.729 |  | 0.061 |  | 0.188 |  | 0.699 |
| Pola-BR (n=27) | 55.3% (29.7-74.8) |  | 57.3% (36.0-73.9) |  | 35.3% (17.1-54.0) |  | 7.4% (1.2-21.4) |  |
| BR (n=18) | 61.6% (25.1-84.4) |  | 35.9% (10.4-62.9) |  | 53.0% (18.1-79.0) |  | 11.1% (1.7-30.4) |  |
| Others (n=19) | 31.6% (7.2-60.3) |  | 21.1% (6.6-41.0) |  | 63.2% (36.2-81.3) |  | 15.8% (3.6-36.0) |  |
| **Response to Bridge chemotherapy** |  | <0.001 |  | <0.001 |  | 0.002 |  | 0.051 |
| CR/PR (n=26) | 81.5% (43.5-95.1) |  | 74.6% (47.1-89.3) |  | 25.4% (7.9-47.8) |  | 0% |  |
| SD/PD (n=38) | 23.8% (6.3-47.5) |  | 19.5% (8.6-33.8) |  | 61.8% (43.4-75.8) |  | 18.6% (8.0-32.7) |  |
| **CRS** |  | 0.929 |  | 0.679 |  | 0.579 |  | 0.199 |
| No (n=23) | 28.3% (1.9-67.0) |  | 43.6% (22.1-63.3) |  | 52.1% (28.0-71.6) |  | 4.3% (0.3-18.7) |  |
| Yes (n=56) | 45.4% (21.5-66.7) |  | 37.6% (22.8-52.3) |  | 47.7% (33.2-64.6) |  | 14.8% (6.8-25.7) |  |
| **ICANS** |  | 0.002 |  | 0.011 |  | 0.820 |  | <0.001 |
| No (n=62) | 49.6% (23.8-71.1) |  | 46.1% (32.1-59.0) |  | 49.0% (34.6-62.0) |  | 4.8% (1.3-12.3) |  |
| Yes (n=17) | 18.3% (1.5-50.5) |  | 14.7% (1.3-42.9) |  | 50.0% (14.4-78.1) |  | 35.3% (13.6-58.0) |  |
| **Tocilizumab usage** |  | 0.571 |  | 0.518 |  | 0.983 |  | 0.339 |
| No (n=47) | 39.0% (9.9-68.2) |  | 42.7% (27.7-57.0) |  | 48.8% (32.7-63.0) |  | 8.5% (2.7-18.7) |  |
| Yes (n=32) | 41.7% (14.1-67.6) |  | 33.6% (12.7-56.1) |  | 49.4% (22.2-71.9) |  | 17.0% (5.8-33.1) |  |
| **Dexamethasone usage** |  | <0.001 |  | 0.004 |  | 0.261 |  | 0.102 |
| No (n=66) | 43.7% (17.9-67.1) |  | 44.1% (29.7-57.6) |  | 46.4% (31.5-60.0) |  | 9.5% (3.8-18.4) |  |
| Yes (n=11) | 15.2% (0.9-47.0) |  | 18.2% (2.9-44.2) |  | 54.5% (19.4-80.0) |  | 27.3% (5.8-55.3) |  |
| **3-month response after CAR T (n=59)** |  | <0.001 |  | <0.001 |  | <0.001 |  | 0.414 |
| Non-CR (n=21) | 28.6% (7.6-54.4) |  | 9.5% (1.6-26.1) |  | 90.5% (61.2-98.0) |  | 0% |  |
| CR (n=38) | 68.3% (26.4-89.7) |  | 77.8% (52.7-90.6) |  | 18.7% (5.0-39.1) |  | 3.4% (0.2-15.2) |  |

CIR, cumulative incidence of relapse; HPI, *Helicobacter pylori* infection; MALT-IPI, mucosa-associated lymphoid tissue lymphoma-International Prognostic Index; NRM, non-relapsed mortality; OS, overall survival; PFS, progression-free survival

† Univariate analysis variables were selected based on prior literature on the currently known prognostic factors.

* Among 151 HPI-positive patients, 7 (4.6%) were diagnosed with Lugano stage II2, IIE, and IV and received chemotherapy

**Supplementary Table 3. Comparison of clinical outcomes for Tisagenlecleucel (Tisa-cel) across global real-world and clinical trial cohorts in relapsed/refractory DLBCL**

| **Location** | **Phase II JULIET^1,2^** | | **CIBMTR^3^** | **Meta-analysis^4^** | **San Diego^5^** | **Japan^6^** | **SMC^7^** | **CMC** |
| --- | --- | --- | --- | --- | --- | --- | --- | --- |
| **Type of CAR T** | Tisa-cel | Tisa-cel | Tise-cel | Tisa-cel | Tisa-cel or Axi-cel | Tisa-cel | Tisa-cel | Tisa-cel |
| **Median f/u*** | 14 months | 40 months | 24.0 months | 16.0 months | 16.3 months | 6.6 months | 17.3 months | 11.6 months |
| **Patients number** | 93-115 patients | | 1159 patients | 1649 patients | 66 patients  (Axi 59, Tisa 7) | 89 patients | 96 patients | 79 patients |
| **Median age** | 56 years | | ≥65 years (57.6%) | 64.3 years | 59.5 years | 59 years | 63 years | 61 years |
| **Previous Tx** | <3 lines: 49% | | ≥3L (61.0%) | Median 3 lines (2.6-3.5) | Median 3L  (range 1-7) | 2L (16.9%), 3L (32.6%), 4L (29.2%), 5L+ (21.3%) | Median 2 lines (2–6) | Median 2 lines (2–7) |
| **ORR (%)** | 52% | 53% | 59.5% | 57.7% | 67% | 73% | 71.9% | 72.9% |
| **CR (%)** | 40% | 39% | 44.5% | 39.0% | 53% | 55% | 45.8% | 64.4% |
| **Median PFS** | NR | 2.9 months | 2yrs-PFS 28.4% | 3.3 months | 10.3 months | 1yrs-EFS 67.0% | 4.3 months  1-year PFS 33.3% | 4.9 months  1-year PFS 42.7% |
| **Median OS** | 12.0 months | 11 months | 2yrs-OS 43.6% | 11.7 months | 28.4 months | 1yrs-OS 46.3% | 13.9 months  1-year OS 55.2% | 21.6 months  1-year OS 59.8% |
| **CRS (All/Grade 3-4)** | 58%/22% | | 58.2%/6.0% | 70.6%/8.9% | 88%/Not Reported | 89.9%/6.7% | 75%/14.6% | 70.9%/22.8% |
| **ICANS (All/Grade 3-4)** | 21%/12% | | 22.5%/7.4% | 19.9%/5.8% | 56%/Not Reported | 5.6%/1.1% | 22.9%/7.3% | 21.5%/8.8% |

Axi-cel, axicabtagene ciloleucel; CAR T-cell, chimeric antigen receptor T-cell therapy; CIBMTR, Center for International Blood and Marrow Transplant Research; CMC, Catholic Medical Center; CR, complete response; CRS, cytokine release syndrome; DLBCL, diffuse large B-cell lymphoma; EFS, event-free survival; f/u, follow-up; ICANS, immune effector cell–associated neurotoxicity syndrome; ORR, overall response rate; OS, overall survival; PFS, progression-free survival; SMC, Samsung Medical Center; Tisa-cel, tisagenlecleucel; Tx, treatment

(1) NEJM 2019;380:45-56.; (2) Lancet Oncol. 2021;22:1403-15.; (3) J Immunother Cancer. 2025;13:e009890.; (4) Transplant Cell Ther. 2024;30:77 e1-77 e15.; (5) Cancers (Basel). 2023;15:4671.; (6) Int J Clin Oncol. 2023;28:816-26.; (7) Int J Clin Oncol. 2025;122:533-45.

* as of data cut-off
